# Extracellular Matrix Defects in Aneurysmal Fibulin-4 Mice Predispose to Lung Emphysema

**DOI:** 10.1371/journal.pone.0106054

**Published:** 2014-09-25

**Authors:** Natasja W. M. Ramnath, Koen M. van de Luijtgaarden, Ingrid van der Pluijm, Menno van Nimwegen, Paula M. van Heijningen, Sigrid M. A. Swagemakers, Bibi S. van Thiel, Ruziedi Y. Ridwan, Nicole van Vliet, Marcel Vermeij, Luuk J. A. C. Hawinkels, Anne de Munck, Oleh Dzyubachyk, Erik Meijering, Peter van der Spek, Robbert Rottier, Hiromi Yanagisawa, Rudi W. Hendriks, Roland Kanaar, Ellen V. Rouwet, Alex Kleinjan, Jeroen Essers

**Affiliations:** 1 Department of Genetics, Cancer Genomics Centre, Erasmus MC, Rotterdam, The Netherlands; 2 Department of Pulmonary Diseases, Erasmus MC, Rotterdam, The Netherlands; 3 Department of Bioinformatics, Erasmus MC, Rotterdam, The Netherlands; 4 Department of Pathology, Erasmus MC, Rotterdam, The Netherlands; 5 Department of Medical Informatics and Department of Radiology, Erasmus MC, Rotterdam, The Netherlands; 6 Department of Radiation Oncology, Erasmus MC, Rotterdam, The Netherlands; 7 Department of Vascular Surgery, Erasmus MC, Rotterdam, The Netherlands; 8 Department of Pharmacology, Erasmus MC, Rotterdam, The Netherlands; 9 Department of Molecular Cell Biology and Centre for Biomedical Genetics, Leiden, The Netherlands; 10 Department of Pediatric Surgery, Erasmus MC, Rotterdam, The Netherlands; 11 Department of Radiology, Leiden University Medical Centre, Leiden, The Netherlands; 12 Department of Molecular Biology, University of Texas Southwestern Medical Center, Dallas, Texas, United States of America; Scuola Superiore Sant'Anna, Italy

## Abstract

**Background:**

In this study we set out to investigate the clinically observed relationship between chronic obstructive pulmonary disease (COPD) and aortic aneurysms. We tested the hypothesis that an inherited deficiency of connective tissue might play a role in the combined development of pulmonary emphysema and vascular disease.

**Methods:**

We first determined the prevalence of chronic obstructive pulmonary disease in a clinical cohort of aortic aneurysms patients and arterial occlusive disease patients. Subsequently, we used a combined approach comprising pathological, functional, molecular imaging, immunological and gene expression analysis to reveal the sequence of events that culminates in pulmonary emphysema in aneurysmal Fibulin-4 deficient (Fibulin-4^R^) mice.

**Results:**

Here we show that COPD is significantly more prevalent in aneurysm patients compared to arterial occlusive disease patients, independent of smoking, other clinical risk factors and inflammation. In addition, we demonstrate that aneurysmal Fibulin-4^R/R^ mice display severe developmental lung emphysema, whereas Fibulin-4^+/R^ mice acquire alveolar breakdown with age and upon infectious stress. This vicious circle is further exacerbated by the diminished antiprotease capacity of the lungs and ultimately results in the development of pulmonary emphysema.

**Conclusions:**

Our experimental data identify genetic susceptibility to extracellular matrix degradation and secondary inflammation as the common mechanisms in both COPD and aneurysm formation.

## Introduction

Chronic obstructive pulmonary disease (COPD) is worldwide one of the major causes of morbidity and mortality [1]. In addition to chronic airflow obstruction due to airway inflammation and alveolar destruction, COPD is associated with extrapulmonary manifestations, including cardiovascular diseases [2]–[5]. These comorbid conditions contribute to the overall disability of patients and complicate the management of COPD.

Aortic aneurysm (AA) is one of the cardiovascular diseases related to COPD [6], [7]. The nature of this relationship is currently unknown. Patients with COPD, AA, and/or atherosclerosis share a number of risk factors, including age, hypertension, and tobacco smoking [8], [9]. Resemblances in risk profiles between these conditions, most notably smoking, may account for the relation between AA and COPD. Furthermore, a systemic inflammatory response has been suggested as a common denominator [10].

The association between COPD and AA prompted us to investigate the prevalence of COPD in a large cohort of patients with aneurysmal or arterial occlusive disease (AOD) in relation to their clinical risk profiles. Here, we found that COPD is much more prevalent in patients with AA compared to those with AOD, irrespective of common clinical risk factors. Since AA [11], [12] and COPD [13] are associated with destruction of the extracellular matrix (ECM), we hypothesized that a primary ECM defect may provide a common ground for the combined development of COPD and aneurysm formation. We previously demonstrated that mice with reduced expression of the ECM glycoprotein Fibulin-4 exhibit ECM degradation in the aortic wall and AA formation [14], [15]. We here investigated the role of Fibulin-4 deficiency in the development of lung emphysema.

## Results

### Clinical study

#### Patient characteristics

We included 1393 patients; 614 patients (44%) were diagnosed with AA and 779 patients (56%) with AOD. The majority of AA patients were treated for an abdominal aortic aneurysm (AAA). None of the patients in this series were treated for an aneurysm of the ascending aorta; 62/614 (10%) of patients were treated for an aneurysm of the descending thoracic aorta (TAA). Clinical characteristics of AA and AOD patients are presented in Table 1. Patients with AA were on average older and more frequently of male gender. Patients with AOD had higher rates of diabetes, hypercholesterolemia, and cerebrovascular disease. In addition, there were differences in medication use between the two groups: statins and antiplatelet drugs were more commonly used by patients with AOD, whereas beta-blockers were more often used by patients with AA. Importantly, smoking rates were similar in the two patient groups. A differentiation in clinical characteristics between AAA and TAA patients is presented in Table S1.

**Table 1:** Clinical characteristics of patients with aortic aneurysm (AA) or arterial occlusive disease (AOD).

	AA	AOD	P-value
	n = 614	n = 779	
Baseline characteristics			
Male gender (%)	525 (85.5)	521 (66.9)	<0.001
Age (years ± SD)	71.4±7.8	65.6±11.0	<0.001
Body mass index (kg/m^2^, mean ± SD)	26.1±3.9	26.2±4.3	0.540
Cardiovascular comorbidities (%)			
Congestive heart failure	66 (10.7)	89 (11.4)	0.692
Ischemic heart disease	272 (44.3)	306 (39.3)	0.059
Cerebrovascular disease	89 (14.5)	366 (47.0)	<0.001
Cardiovascular risk factors (%)			
Kidney disease	94 (15.3)	106 (13.6)	0.368
Diabetes mellitus	103 (16.8)	225 (28.9)	<0.001
Hypertension	408 (66.4)	524 (67.2)	0.761
Hypercholesterolemia	534 (87.0)	706 (90.6)	0.030
Smoking – current	236 (38.4)	338 (43.3)	0.068
Smoking – ever	473 (77.0)	613 (78.7)	0.459
Medication (%)			
Statins	446 (72.6)	633 (81.2)	<0.001
Beta-blockers	531 (86.4)	592 (75.9)	<0.001
Renin-angiotensin system inhibitors	271 (44.1)	369 (47.3)	0.247
Diuretics	138 (22.4)	211 (27.0)	0.052
Antiplatelets	353 (57.4)	581 (74.5)	<0.001

#### Association between COPD and AA

**Figure 1:**
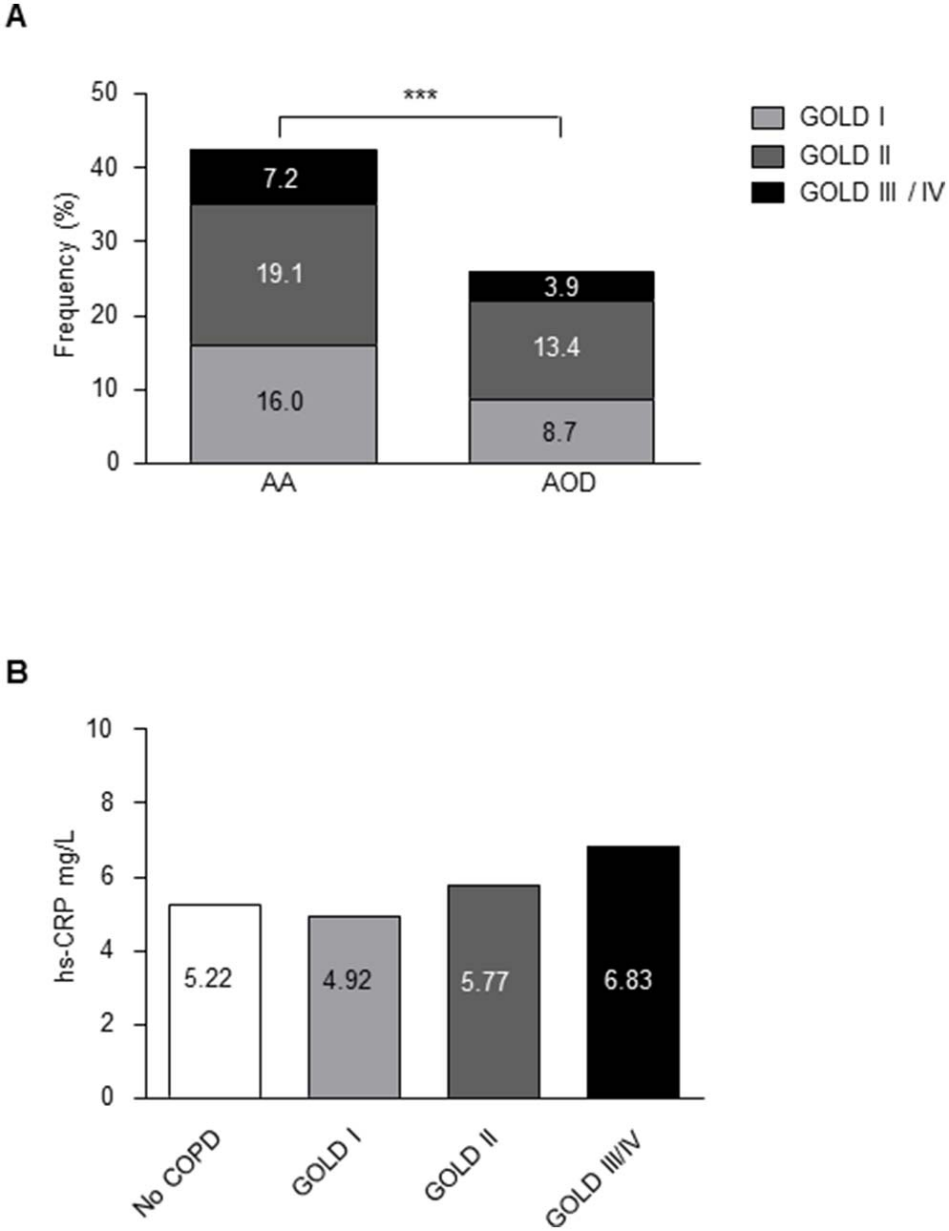
Prevalence and severity of COPD in patients with an aortic aneurysm (AA) or arterial occlusive disease (AOD). (A) The prevalence of COPD in all GOLD classes was higher in AA (n = 614) compared to AOD patients (n = 779, ***p<0.001). (B) Serum high-sensitivity CRP levels according to severity of COPD in patients with AA or AOD. There was no significant difference between patients with and without COPD (p for trend = 0.123).

COPD was more common in AA patients as compared to AOD patients (42% vs. 26%, p<0.001, Figure 1A). COPD rates did not differ between TAA and AAA patients (Table S2). Univariate logistic regression analysis showed a significant association between COPD and AA (odds ratio 2.08, 95%CI: 1.66–2.61, p<0.001; Table S3). Since patients with COPD, AA, and AOD shared a number of cardiovascular risk factors, we subsequently performed a multivariable regression analysis. Even after adjustment for potentially confounding factors the association between COPD and AA remained significant (odds ratio 1.56, 95%CI: 1.16–2.10, p = 0.003; Table S3).

As inflammation is involved in aneurysm development, atherosclerosis, and COPD, we measured serum levels of the systemic inflammatory biomarker high-sensitivity C-reactive protein (hs-CRP). The median serum hs-CRP concentration was higher in patients with AA compared to AOD (5.9 [IQR 2.9–12.5] vs 4.8 mg/L [IQR 2.1–11.1], p = 0.02). However, there were no differences in hs-CRP levels between arterial disease patients with COPD and those without COPD (median 5.4 vs 5.2 mg/L, p = 0.776; Figure 1B). These data strongly support the association between AA and COPD in patients independently of smoking and other cardiovascular risk factors.

### Experimental study

#### Extracellular matrix remodeling in aortas of Fibulin-4 deficient mice

**Figure 2:**
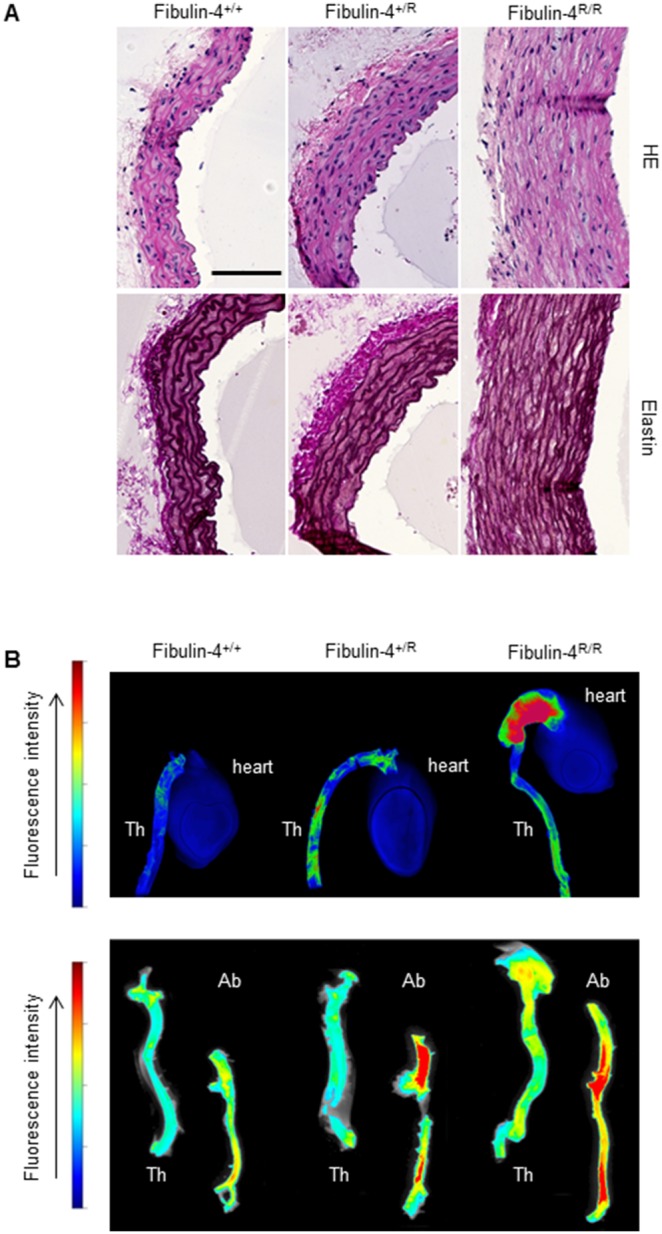
Degenerative arterial wall changes and increased MMP activity in Fibulin-4 animals. (A) Comparison of the architecture of the aortic wall in Fibulin-4^+/+^, Fibulin-4^+/R^, and Fibulin-4^R/R^ mice. Top: Haematoxylin- eosin (HE) staining of cross-sections from 120 day-old mice. Bottom: Aberrations in elastic laminae in Fibulin-4^R/R^ mice, consisting of a fragmented and disorganized appearance of elastin in the medial layers of the aorta. In heterozygous Fibulin-4^+/R^ mice, interrupted elastin layers and areas in the aortic wall with a granular appearance of elastic laminae are present, albeit milder, indicating a gene dosage effect. Scale bar indicates 100 µm (B) Ex-vivo analysis shows a gradual increase in active MMPs as detected with the activatable NIRF probe MMPSense 680. Within the aneurysmal lesions in the thoracic aorta (Th), as well as in the abdominal aorta (Ab) of Fibulin-4^+/R^ and Fibulin-4^R/R^ mice, a graded increase in MMP activity is detected.

Complete disruption of Fibulin-4 is incompatible with life as targeted disruption of Fibulin-4 abolishes elastogenesis and causes perinatal lethality in mice [16]. We previously generated a viable mouse model for Fibulin-4 using a hypomorphic Fibulin-4 allele (Fibulin4^R^) which results in reduced expression by transcriptional interference through placement of a TKneo targeting construct in a downstream gene (Mus81)[14]. While Mus81 knockout mice, from which the selectable marker was removed, were born at expected Mendelian frequencies and were indistinguishable from wild type littermates in terms of development, growth, immune function and fertility [17], our Fibulin-4 hypomorphic mice displayed a 2-fold lower expression of Fibulin-4 in heterozygous Fibulin-4^+/R^ aortas and a 4-fold downregulation in homozygous Fibulin-4^R/R^ aortas, resulting in ECM defects and vascular abnormalities, including aortic aneurysms in the aorta ascendens [14]. Indeed, comparison of haematoxylin-eosin (HE) and elastin stained aortas of 3-month old Fibulin-4^+/+^, Fibulin-4^+/R^ and Fibulin-4^R/R^ mice showed severe thickening and decellularization of the medial layer of the ascending aortic wall in Fibulin-4^R/R^ mice and fragmented and disorganized elastin laminae resulting in 2–3 fold dilated ascending aortic aneurysms in all homozygous knockdown animals analyzed (Figure 2A). While Fibulin-4^+/R^ aortas are not dilated, careful histological comparison showed an increased medial thickness, signs of elastin breakage and increased deposition of amorphous cell material between the elastin layers compared to wild type animals (Figure 2A). The downregulation of Fibulin-4 leads to elastin abnormalities in the ascending aorta accompanied by extensive remodeling of the ECM presumably through activation of matrixmetalloproteinases (MMPs). The increased activity of MMPs can be visualized with an MMP-specific activatable near-infrared (NIRF) probe developed for *in vivo* imaging (Perkin Elmer). Fibulin-4^+/R^ and Fibulin-4^R/R^ mice injected with this protease sensing probe show a gradual increase in NIRF signal in the thoracic area, indicative of aneurysm formation [14], [15]. Here, we injected this MMP activatable NIRF probe and sacrificed the mice after 24 hrs after which the hearts with aortas were excised. When we compared the *ex vivo* fluorescence in the aortic arch and descending aorta using the Odyssey imaging system, we measured a gradual increase of MMP activation in Fibulin-4^+/R^ and Fibulin-4^R/R^ compared to wild type Fibulin-4^+/+^ mice (Figure 2B, upper row). Since this was consistent with the gradual elastin degradation we noticed histologically, we then used these protease sensing probes to analyze the abdominal part of the aorta, below the diaphragm. *Ex-vivo* analysis confirmed the *in vivo* observed gradual increase in MMPs within the aneurysmal lesions in the thoracic aorta (Th) and showed as well a gradual increased activity in the abdominal aorta (Ab) in both Fibulin-4^+/R^ and Fibulin-4^R/R^ mice. Thus, decreased expression of fibulin-4 not only leads to MMP activation in the thoracic part of the aorta, but equally affects the abdominal aorta, predisposing the complete aorta for arterial disease. Since we find extracellular matrix remodeling activity in both thoracic and abdominal aorta, we conclude that the Fibulin-4^+/R^ and Fibulin-4^R/R^ mouse models mimics different stages of both TAA and AAA.

#### Alveolar airspace enlargement in Fibulin-4 deficient mice

In the clinical study, we observed a significant association between COPD and AA. To investigate whether ECM abnormalities may provide a common ground for aneurysm formation and COPD we subsequently analyzed whether fibulin-4 deficiency also predisposes for lung abnormalities in these Fibulin-4 hypomorfic mice.

**Figure 3:**
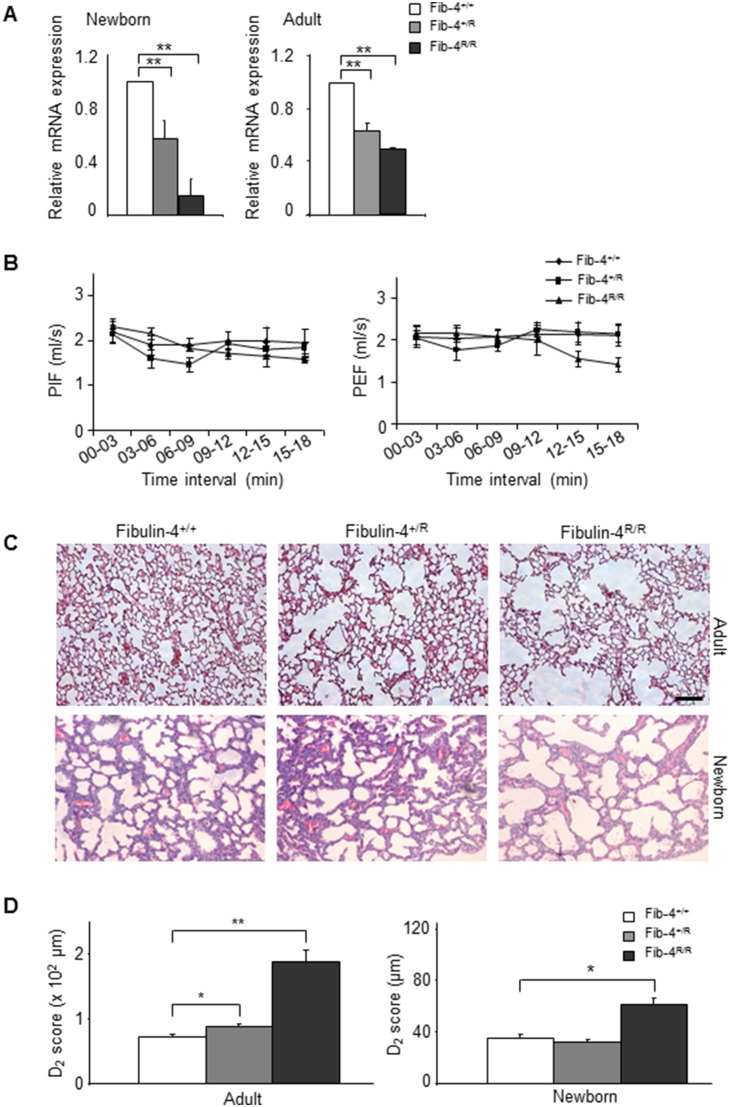
Enlarged alveolar airspaces in lungs of Fibulin-4 knockdown mice. (A) Expression levels of Fibulin-4 in lungs isolated from newborn (n = 4, n = 4, n = 3) and adult (n = 4, n = 4, n = 4) Fibulin-4^+/+^, Fibulin-4^+/R^ and Fibulin-4^R/R^ mice relative to Fibulin-4^+/+^ lungs (**p<0.01). (B) Mean peak inspiratory flow (PIF) and peak expiratory flow (PEF) values for Fibulin-4^+/+^ (n = 4), Fibulin-4^+/R^ (n = 4) and Fibulin-4^R/R^ mice (observed for n = 4, but two animals died during the procedure) at 3-minute intervals. After a 9 minute adaptation period (the first three time intervals), PIF follows similar trends in Fibulin-4^+/+^, Fibulin-4^+/R^ and Fibulin-4^R/R^ mice, while Fibulin-4^R/R^ mice show a decrease in PEF. (C) HE stained sections of formalin fixed lungs of male mice. Enlarged alveolar airspaces are observed in Fibulin-4^+/R^ (middle, n = 3) and Fibulin-4^R/R^ lungs (right, n = 3), with the latter being more pronounced, compared to Fibulin-4^+/+^ (n = 3). Enlarged alveolar airspaces are already present in Fibulin-4^R/R^ newborn lungs (n = 3), while lungs of Fibulin-4^+/R^ littermates (n = 5) show no difference compared to Fibulin-4^+/+^ lungs (n = 4). Scale bar 100 µm. Magnification 10x. (D) D_2_ quantification (see methods and Figure S1 for further explanation) of the alveolar airspaces revealed a significant difference between adult Fibulin-4^+/+^ and Fibulin-4^+/R^ (*p<0.05) and between adult Fibulin-4^+/+^ and Fibulin-4^R/R^ lungs (**p<0.01) as well as between newborn Fibulin-4^+/+^ and Fibulin-4^R/R^ lungs (*p<0.05).

To this end, we first tested whether the transcriptional downregulation of Fibulin-4 also occurs in the lungs of these mutant mice. Expression levels of Fibulin-4 mRNA in newborn and adult lungs of Fibulin-4^+/R^ and Fibulin-4^R/R^ mice were indeed significantly lower compared to Fibulin-4^+/+^ mice (Figure 3A). Next, we examined whether Fibulin-4 animals display lung emphysema. Assessment of respiratory performance by whole-body plethysmography showed similar breathing frequencies and Peak Inspiratory Flows (PIF) in adult Fibulin-4 deficient mice and Fibulin-4^+/+^ littermates, whereas the Peak Expiratory Flow (PEF) tended to decrease over time in Fibulin-4^R/R^ mice (Figure 3B). Significance could not be determined since 2 out of 4 Fibulin-4^R/R^ mice died during the course of the experiment.

Downregulation of Fibulin-4 was accompanied by alveolar airspace enlargement in adult Fibulin-4^+/R^ and Fibulin-4^R/R^ lungs (Figure 3C). In newborn mice, reduced pulmonary Fibulin-4 expression levels coincided with clear alveolar airspace enlargements in Fibulin-4^R/R^ lungs, but not in Fibulin-4^+/R^ lungs (Figures 3C and 3D, and accompanying Figure S1A and B). Importantly, analysis of the aortas of the adult mice used for lung analysis showed a gradual thickening of the medial layers of the aorta (Figure S1C). Elastin staining showed sites of complete fragmentation and disarray of the elastin layers in Fibulin-4^R/R^ aortas and increased deposition of amorphous material between the elastin layers in Fibulin-4^+/R^ animals (Figure S1D), indicating that a similar gene-doses decrease in Fibulin-4 expression affects both lungs and the aortic wall in these mice. Immunohistochemistry on lung tissue with antibodies specific for certain lung cell markers, including thyroid transcription factor 1 (TTF-1), Clara-cell-specific protein (CC-10), and α-smooth muscle actin (α-SMA) demonstrated no differences in the presence and relative distribution of the major cell types in the lungs of Fibulin-4^+/R^ and Fibulin-4^R/R^ mice (Figure S2), which may exclude altered airway-cell differentiation.

These results show that in addition to aortic abnormalities, a decrease in Fibulin-4 expression leads to gene dose-dependent alterations in the lung. While the emphysematous changes in the lungs of newborn Fibulin-4^R/R^ mice suggest a developmental defect, Fibulin-4^+/R^ mice acquired the COPD phenotype with age.

#### Transcriptome analysis of Fibulin-4^+/R^ and Fibulin-4^R/R^ lungs

In order to get an idea of the underlying processes involved, we performed gene expression analysis on mRNA isolated from Fibulin-4^+/+^, Fibulin-4^+/R^ and Fibulin-4^R/R^ lung tissue, both newborn as well as adult. Comparison of RNA expression between newborn Fibulin-4^+/+^, Fibulin-4^+/R^ and Fibulin-4^R/R^ lungs with Significance Analysis of Microarrays [18] revealed a limited set of differentially regulated genes. Comparison of RNA expression between adult Fibulin-4^+/+^ and Fibulin-4^R/R^ lungs using SAM (FDR 10%) revealed 374 deregulated genes (both up- and downregulated), whereas no deregulated genes were found between Fibulin-4^+/+^ and Fibulin-4^+/R^ lungs.

Of the 20 most significantly up-regulated genes in adult Fibulin-4^R/R^ lungs, 50% were involved in inflammation processes (Table 2 and Table S4). Network analysis with Ingenuity pathway analysis (IPA) on the 374 deregulated probes revealed many significantly changed pathways involved in the immune system (Table S5). This suggests that the severe airspace enlargement in adult Fibulin-4^R/R^ lungs coincides with overexpression of genes involved in inflammatory processes.

**Table 2:** The most significantly up-regulated genes in adult Fibulin-4^R/R^ lungs. Genes are indicated with their ratios compared to Fibulin-4^+/+^ lungs and the process involved.

Top up-regulated genes		
Genes	Ratio	Function
Arg1	3.68	Urea cycle
Slpi	3.37	Inhibitor serine proteases
Ms4a4b	2.38	T-cell regulation
Wisp2	2.26	Inhibits proliferation of vascular smooth muscle cells
Prkcb	2.03	B-cell activation, apoptosis
Emr4	1.98	Mediate between myeloid- and B-cells
Cd300a	1.92	Leukocyte cell surface proteins
Gzma	1.93	Cytotoxic T-cell and natural killer cell specific serine proteases
Klra4	1.91	Natural killer cell receptor
Nkg7	1.90	Natural killer cell granule protein
Ctsw	1.84	Regulation of T-cell cytolytic activity
Wisp1	1.83	Matrix remodeling
Lrat	1.64	Retinoid cycle
Plac8	1.81	Defense response to bacterium
Ccl5	1.78	Chemotactic cytokine and plays active role in recruiting leukocytes
Tspan32	1.73	Tumor suppressing fragment
Cyp51a1	1.70	Production of sterols
Rbm3	1.68	Temperature induced
Bcl2	1.66	Apoptosis regulator
Mef2c	1.65	Transcription factor important for vascular development

#### Spontaneous inflammation in adult Fibulin-4^R/R^ lungs

**Figure 4:**
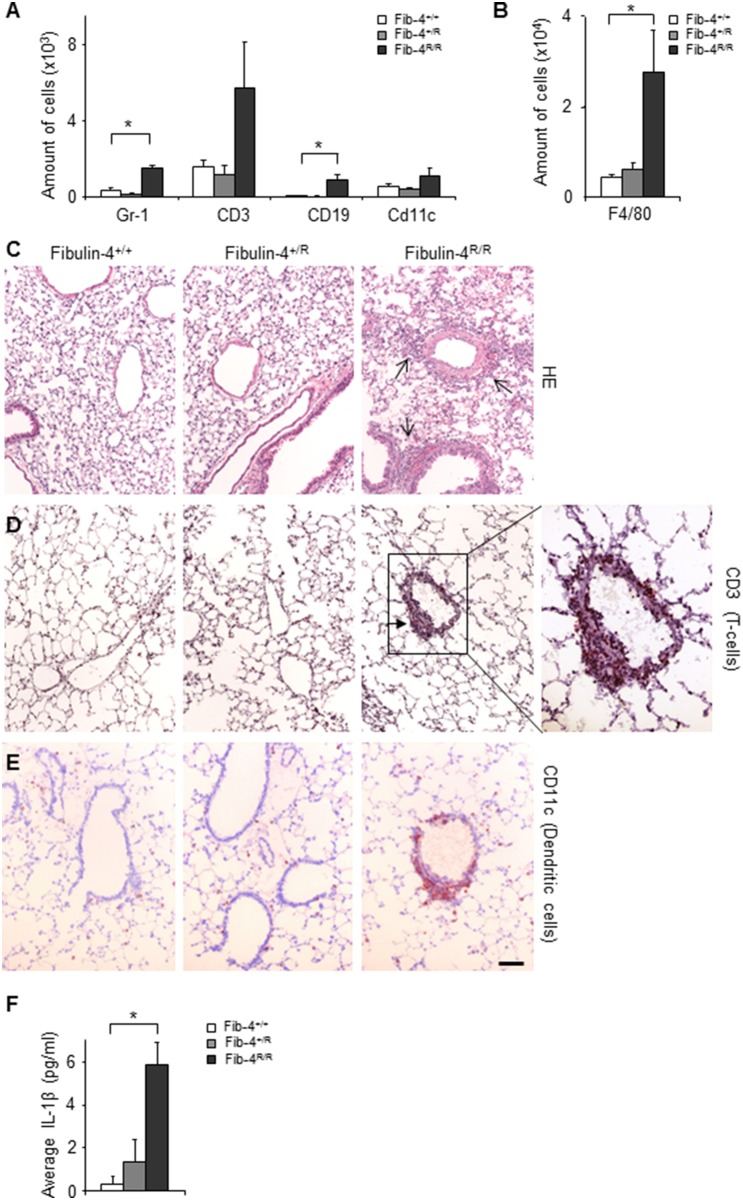
Increased inflammation in Fibulin-4^R/R^ lungs compared to Fibulin-4^+/+^ and Fibulin-4^+/R^ lungs. (A) Flow cytometric analysis revealed more Gr1+ granulocytes and CD19+ B-cells in BAL samples from Fibulin-4^R/R^ (n = 4) compared to Fibulin-4^+/+^ mice (n = 4, *p<0.05) and (B) increased numbers of F4/80 macrophages in Fibulin-4^R/R^ lungs (n = 4, *p<0.05). (C) HE stained sections from adult (n = 4, n = 4, n = 4) Fibulin-4^+/+^, Fibulin-4^+/R^ and Fibulin-4^R/R^ lungs showing focal infiltrations around vessels and airways in Fibulin-4^R/R^ lungs (black arrows). (D) Staining for T-cells (CD3+) and (E) dendritic cells (CD11c+) points to the presence of inflammatory cells within the focal infiltrations. Magnification 20x. Scale bar 50 µm. (F) ELISA analysis showing increased IL-1β levels in Fibulin-4^R/R^ lungs (n = 4, *p<0.05).

To investigate whether indeed the immune system shows significant alterations in the lungs of Fibulin-4^R/R^ animals, we did flow cytometric analysis of broncho-alveolar lavage (BAL) samples. This analysis showed more inflammatory cells, in particular granulocytes (Gr-1+) and B-cells (CD19+), in lungs of adult Fibulin-4^R/R^ compared to Fibulin-4^+/+^ and Fibulin-4^+/R^ mice (Figure 4A). Cell suspensions of Fibulin-4^R/R^ lungs contained significantly more macrophages (F4/80+) compared to Fibulin-4^+/+^ and Fibulin-4^+/R^ lungs (Figure 4B), and tended to contain more T-cells (CD3+) and dendritic cells (CD11c+). Immunohistochemical analysis showed focal infiltrations of inflammatory cells around veins and bronchi in adult Fibulin-4^R/R^ lungs (Figure 4C), mainly consisting of T-cells and dendritic cells. Cytokine analysis of BAL samples showed significantly higher levels of IL-1β in Fibulin-4^R/R^ but not in Fibulin-4^+/R^ as compared to Fibulin-4^+/+^ mice (Figure 4D) Interestingly, IL-1β is a pro-inflammatory cytokine which is mainly produced by activated macrophages and which is increased in patients with COPD [19], [20]. These data indicate that the severe airspace enlargement observed in lungs of adult Fibulin-4^R/R^ mice was accompanied by up-regulation of inflammatory pathways, whereas the milder lung abnormalities in Fibulin-4^+/R^ animals were not associated with an explicit inflammation process.

#### Disturbed TGF-β signaling in Fibulin-4 deficient lungs

Since degradation of the vascular wall in aortic aneurysms is related to disturbances in the TGF-β signaling pathway [14], [21], we next investigated the role of TGF-β signaling in alveolar wall degradation in Fibulin-4 deficient mice. Although the gene expression analysis in lung mRNA samples only gave rise to a limited set of deregulated genes in newborn Fibulin-4^R/R^ animals, it did reveal downregulation of the Pias4 gene in Fibulin-4^R/R^ compared to Fibulin-4^+/+^ lungs (1.2-fold, p<0.05, Table S6). In adult Fibulin-4^R/R^ lungs we identified up-regulation of TGF-β2 and downregulation of the type 2b activin A receptor. In adult Fibulin-4^+/R^ lungs the ‘SMAD specific E3 ubiquitin protein ligase 1’ (Smurf1) gene was significantly downregulated compared to Fibulin-4^+/+^ lungs.

**Figure 5:**
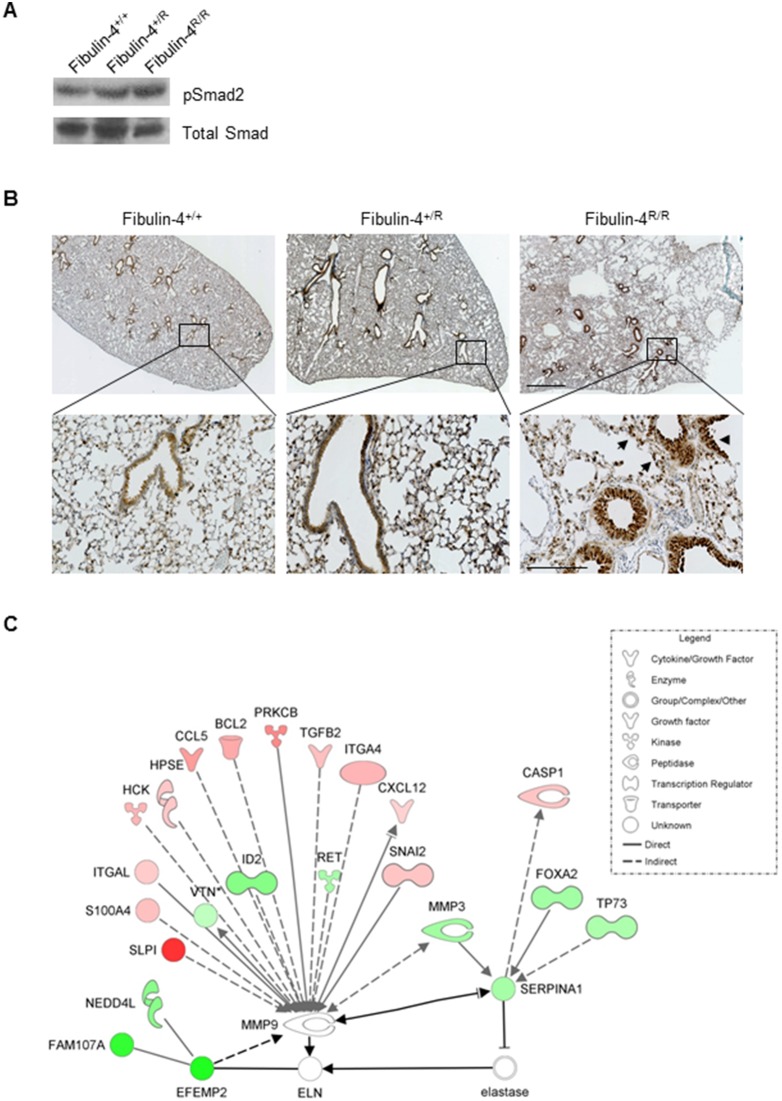
Increased TGF-β signaling in Fibulin-4^+/R^ and Fibulin-4^R/R^ lungs. Immunoblot analysis of pSmad2 in lung homogenates shows an increase in the amount of pSmad2 (A) and pSmad3 (B) in Fibulin-4^+/R^ (n = 3) and Fibulin-4^R/R^ (n = 3) lungs, compared to the total amount of Smad, and to their Fibulin-4^+/+^ control (n = 3). (C) Increased pSmad2 staining of inflammatory and endogenous cells on Fibulin-4^+/R^ (n = 3) and Fibulin-4^R/R^ (n = 3) lung sections. Magnification 2.5x (scale bar 1 mm) upper panel and 20x (scale bar 200 µm) lower panel. (D) Ingenuity pathway explorer showed MMP9 as the shortest connection between Fibulin-4 and SERPINA1. SERPINA1 inhibits neutrophil elastase, which affects elastin. MMP9 itself was not deregulated in Fibulin-4^R/R^ lungs (n = 4), but could be connected to 16 deregulated genes in Fibulin-4^R/R^ compared to Fibulin-4^+/+^ lungs (n = 4, red, up-regulated; green, downregulated), suggestion altered MMP9 activity. Black arrows indicate the connection between Fibulin-4 (EFEMP2), MMP9, SERPINA1, elastase and ELN. Grey arrows indicate the connection of these genes with deregulated genes between Fibulin-4^+/+^ and Fibulin-4^R/R^ lungs.

To check at the protein level whether changes in the TGF-β system occurred, we performed immunoblot analysis for phosphorylation of Smad2 (pSmad2), an intracellular mediator of the TGF-β pathway. These blots showed a gradual increase in pSmad2 in adult Fibulin-4 deficient lungs, indicating increased TGF-β activity (Figure 5A). In Fibulin-4^+/R^ lungs we observed a 1.32-fold change for pSmad2 relative to total Smad and a 1.23-fold change relative to actin when compared to Fibulin-4^+/+^ lungs. In Fibulin-4^R/R^ lungs we observed a 1.67-fold and 1.5-fold change, respectively (Figure 5A and data not shown). Accumulation of phosphorylated smad2 is a general read out of activity for the TGF-β signaling pathway. Smad4 binding to phosphorylated Smad2 is necessary for translocation to the nucleus Subsequently the smad2/3/4 complex can then bind to the DNA after which transcription is initiated. We therefore also determined phosphorylation of pSmad3 in protein extracts of the lungs of Fibulin-4^+/+^, Fibulin-4^+/R^ and Fibulin-4^R/R^ mice and find a gradual increase of pSmad3 in the mutant animals confirming the activation of the central TGF-β transcription factor Smad2/3/4 (Figure 5B). Immunohistochemistry confirmed increased pSmad2 expression in Clara cells lining the bronchioles of the lungs as well as in inflammatory cell infiltrates (Figure 5C). Together these data show that TGF-β activity is mildly increased in adult Fibulin-4^+/R^ and Fibulin-4^R/R^ lungs, which may contribute to the breakdown of alveolar walls in adult Fibulin-4 deficient mice. This upregulation of TGF-β signaling is reminiscent of the upregulation that has been observed in the aortas of both mouse models of aortic aneurysms as well as in patients [14], [15], [27].

#### Overlapping downregulation of SERPINA1 in lungs of Fibulin-4 deficient mice and COPD patients

To investigate a potential common underlying mechanism of the observed lung emphysema phenotype in our Fibulin-4 animals and that in COPD patients, we compared our mouse dataset to gene lists related to COPD that we derived from IPA and gene expression datasets from lung emphysema patients. A search in IPA with the search term ‘chronic obstructive pulmonary disease’ gave 248 records, which we refer to as ‘COPD-related genes’. Next, gene expression data from the comparison between Fibulin-4^+/+^ and Fibulin-4^R/R^ lungs (374 genes) were compared to this list of COPD-related genes derived from IPA, where we found an overlap of 6 genes: PDE3B (1.28 ↑), HCK (1.55 ↑), PRF1 (1.47 ↑), SERPINA1 (1.38 ↓), FGFR3 (1.28 ↓), and EFEMP2 (i.e. Fibulin-4, 3.57 ↓). In a second analysis, we compared the 374 deregulated mouse genes to a list of 125 deregulated genes from the comparison of GEO dataset GSE8581 (1.5-fold, FDR 30%), consisting of 15 COPD cases (predicted FEV1<70%, FEV1/FVC<0.7) and 18 control cases (predicted FEV1>80%, FEV1/FVC>0.7). This comparison showed an overlap of ITPKC (1.28 ↓), KIAA1377 (1.45 ↓), and SERPINA1 (1.38 ↓). Remarkably, these two independent methods both identified SERPINA1 as an overlapping downregulated gene. SERPINA1 encodes for the serine protease inhibitor α-1 antitrypsin, whose targets include elastase. Interestingly, deficiency in α-1 antitrypsin in patients is associated with lung emphysema [22], [23].

In the mouse lung mRNA gene expression analysis SERPINA1 was significantly downregulated in both Fibulin-4^R/R^ lungs (1.38-fold, p<0.01) as well as in Fibulin-4^+/R^ lungs (1.59-fold, p<0.01) compared to Fibulin-4^+/+^ lungs. We used Path explorer in IPA that identifies pathways between differentially expressed genes, in order to determine the relation between Fibulin-4 and SERPINA1. By calculating the shortest path between Fibulin-4 (EFEMP2) and SERPINA1, an indirect connection of Fibulin-4 to MMP9 and a direct connection of MMP9 to SERPINA1 was revealed, as indicated by the black arrows in Figure 5D [14], [24], [25]. Surprisingly, by connecting Fibulin-4, MMP9 and SERPINA1 (both direct and indirect) to the significantly deregulated genes identified in the SAM comparison between Fibulin-4^+/+^ and Fibulin-4^R/R^ lungs (grey arrows), we found an interaction between 16 of those significantly deregulated genes and our dataset with MMP9 (Figure 5C). Importantly, MMPs are proteins involved in remodeling of the ECM, and play a role in aneurysm formation. As we hypothesized that ECM defects provide the link between the relation between AA and COPD, MMPs could very well be part of the underlying mechanism. Since our gene expression analysis did not show a deregulation of MMP9 at the mRNA level, yet the pathway analysis pointed towards involvement of MMP9, we next investigated MMP9 protein activity in Fibulin-4^R/R^ and Fibulin-4^+/R^ lungs.

#### Increased MMP and neutrophil elastase activity in Fibulin-4 deficient lungs

**Figure 6:**
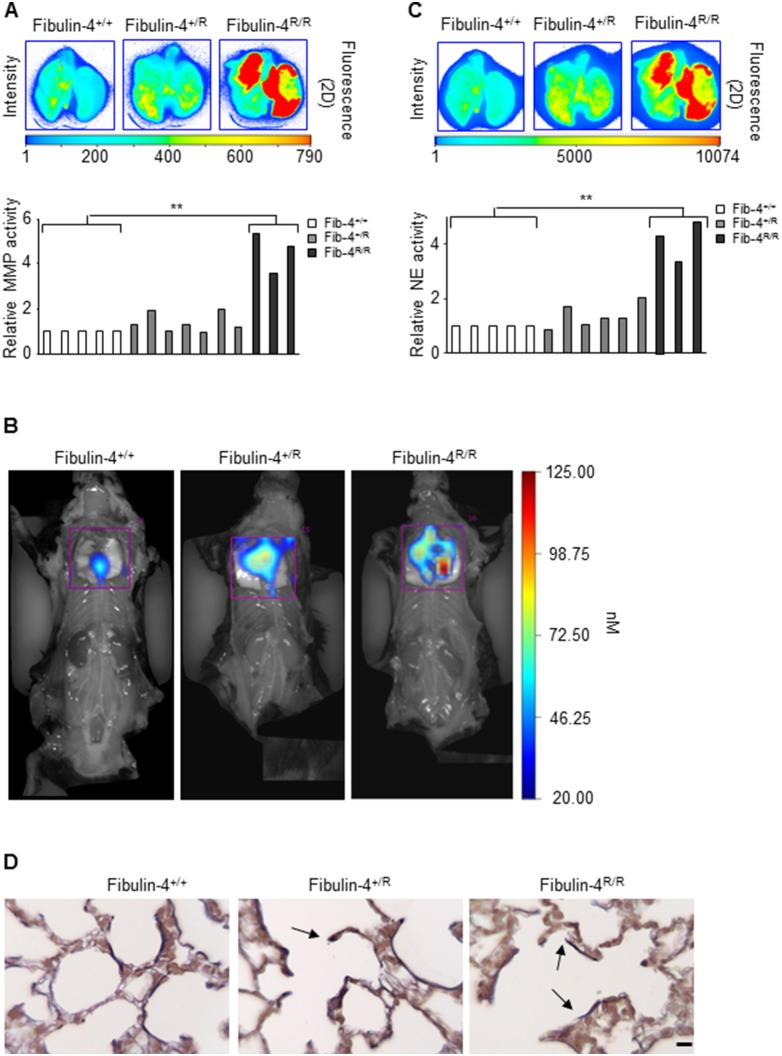
Higher MMP and NE activity in Fibulin-4^+/R^ and Fibulin-4^R/R^ lungs. *In* (A) and (C) *ex vivo* imaging of excised lungs using Odyssey shows increased activity of MMP and NE respectively in Fibulin-4^+/R^ (n = 7) and Fibulin-4^R/R^ lungs (observed for n = 5, but two animals died during the procedure) as compared to Fibulin-4^+/+^ lungs (n = 5), with a significant upregulation for Fibulin-4^R/R^ lungs (**p<0.01). (B) Open-chest registration of NE activity with Neutrophil Elastase FAST 680 probes shows increased activity in Fibulin-4^+/R^ and Fibulin-4^R/R^ lungs as compared to Fibulin-4^+/+^ lungs. (D) Elastin staining of Fibulin-4^+/+^, Fibulin-4^+/R^ and Fibulin-4^R/R^ lungs (n = 3, n = 3, n = 3) shows fragmented elastin layers in Fibulin-4^+/R^ and Fibulin-4^R/R^ lungs, indicated by arrows. Magnification 40x. Scale bar 10 µm.

Fluorescent imaging using protease activatable probes showed increased pulmonary MMP activity in adult Fibulin-4 deficient mice (Figure 6A). The observed decrease in the elastase inhibitor SERPINA1 and the increased MMP activity, which is also associated with cleavage of α-1 antitrypsin [24], might lead to increased activity of neutrophil elastase (NE) (also see Figure 5C for this relation). Indeed, we observed a graded increase in NE activity in adult Fibulin-4^+/R^ and Fibulin-4^R/R^ lungs compared to Fibulin-4^+/+^ lungs (Figures 6B and C). In line with this, pulmonary elastin staining demonstrated interruptions in the elastin layers in Fibulin-4^+/R^ lungs and even more in Fibulin-4^R/R^ lungs compared to those of Fibulin-4^+/+^ animals (Figure 6D), as previously found in the aortas of these mice [14]. Newborn Fibulin-4^R/R^ lungs also displayed elastin abnormalities, while newborn Fibulin-4^+/R^ lungs were comparable to those of Fibulin-4^+/+^ mice (data not shown).

#### Increased and prolonged inflammatory response in lipopolysaccharide (LPS) exposed Fibulin-4^+/R^ mice

**Figure 7:**
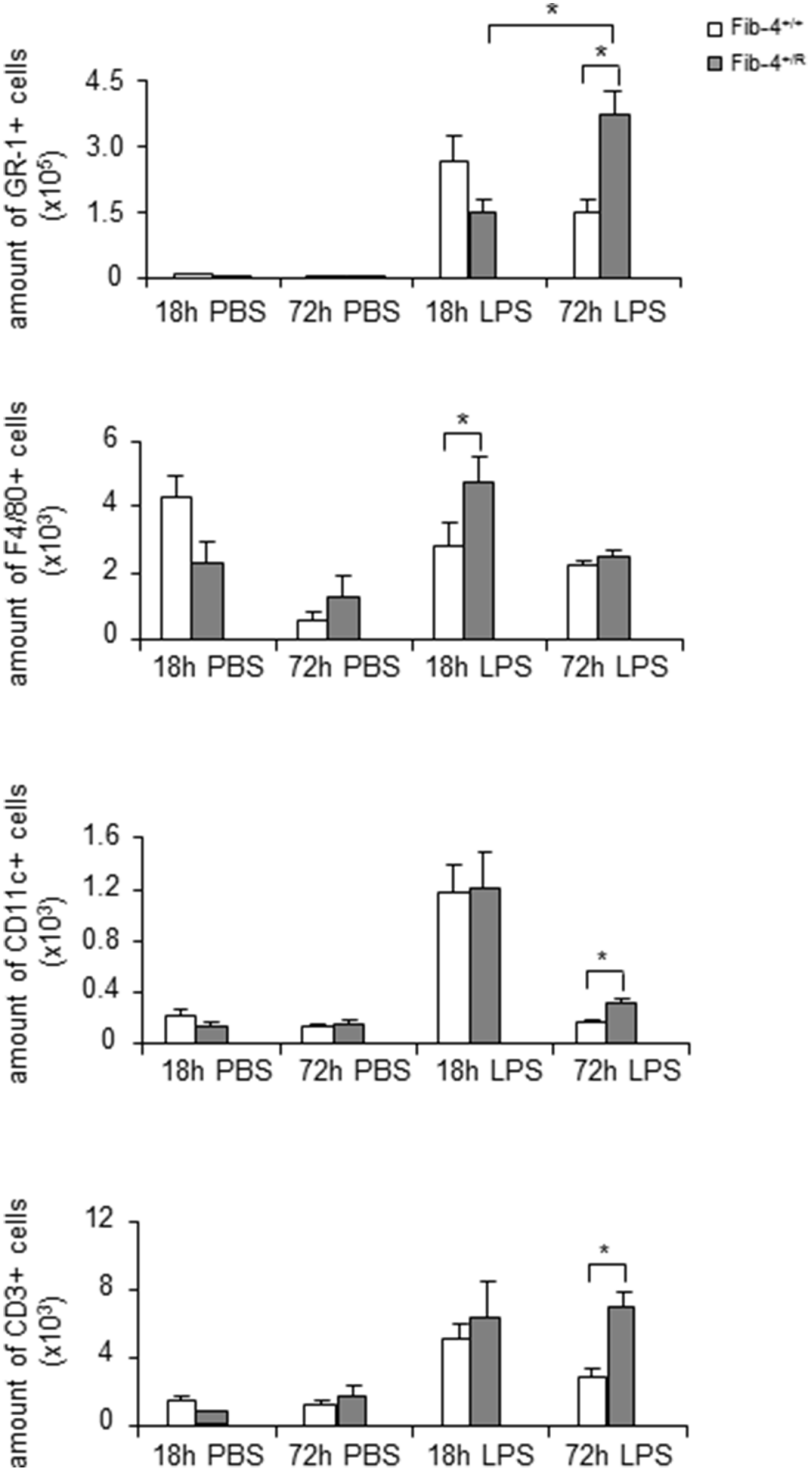
Increased and prolonged inflammatory response in LPS exposed Fibulin-4^+/R^ mice. Quantification of immune cells shows significantly increased F4/80+ cells after 18 hours of LPS exposure in Fibulin-4^+/R^ lungs (n = 4) and a significantly higher number of Gr1+, CD11c+, and CD3+ cells after 72 hours of LPS exposure as compared to Fibulin-4^+/+^ lungs (n = 4, *p<0.05).

Fibulin-4^+/R^ mice developed alveolar airspace enlargement with age together with increased MMP9 and NE activity in the absence of inflammation. However, when adult Fibulin-4^+/R^ lungs were triggered by LPS administration, which mimics bacterial infection in mice by initiating the infiltration of inflammatory cells into the pulmonary alveoli similar to patients with COPD exacerbation [26], [27], flow cytometric analyses of BAL samples and pulmonary cell suspensions showed an increased and prolonged inflammatory response as compared to Fibulin-4^+/+^ mice. There was a significantly greater influx of macrophages (F4/80+) in the lungs 18 hours after LPS exposure and significantly higher numbers of dendritic cells (CD11c+), T-cells (CD3+), and granulocytes (GR1+) 72 hours after LPS exposure (Figure 7 and Figure S3). Moreover, opposite dynamics were observed; in the Fibulin-4^+/+^ lungs the amount of GR1+ cells decreased after 18 hours, while an increase was observed in Fibulin-4^+/R^ lungs 72 hours after LPS exposure. The increase of inflammatory cells after LPS exposure was significantly higher when compared to PBS. The levels of pro-inflammatory cytokines released upon LPS exposure, including IL-1β, TNF-α, and keratinocyte-derived chemokine, were not different between groups (data not shown). These data indicate that Fibulin-4^+/R^ mice exhibit an intensified inflammatory response in the lungs.

## Discussion

In this study we show that COPD is more common in patients with AA than in patients with atherosclerotic arterial disease. This relationship was independent of cigarette smoking and other known risk factors. Furthermore, there was no difference in serum hs-CRP levels between patients with and without COPD, indicating that inflammation per se is unlikely to account for the observed relation between COPD and AA. The findings in this large patient cohort are in line with previous observations of reduced respiratory function in smaller series of AAA patients [6], [7], [10]. Although some previous studies concluded that the association between COPD and aneurysm formation was related to smoking, medication use or presence of other cardiovascular risk factors, these associations became non-significant after correction in multivariable analyses. [28], [29]. Our findings suggest that factors other than cardiovascular risk profiles or systemic inflammation contribute to the association between COPD and AA.

Since both diseases, COPD and AA, are characterized by breakdown of the ECM in the airways and –spaces and in the aortic wall, we investigated whether a primary ECM defect provides the pathogenic link between these two diseases. Analogous to the observed degradation of the aortic wall, up-regulation of MMP activity both thoracic as well as abdominal, and previously observed formation of AAs in mice deficient in the ECM component Fibulin-4, we found that gradual downregulation of Fibulin-4 in the lungs correlated with destruction of alveolar walls and airspace enlargement that is characteristic for lung emphysema. Similar to embryonically lethal, complete Fibulin-4 knockout mice [16], alveolar breakdown was already present in lungs of newborn Fibulin-4^R/R^ mice, and became progressive with age. In contrast, Fibulin-4^+/R^ mice, which have only a 2-fold reduction in the amount of Fibulin-4, had normal elastin structures and alveolar airspaces at birth, but acquired alveolar breakdown with ageing.

Analogous to the activation of the TGF-β pathway in the aortas of Fibulin-4 deficient mice [14], we here demonstrate enhanced activation of the TGF-β pathway in the lungs of Fibulin-4^+/R^ and Fibulin-4^R/R^ mice. The co-occurrence of lung emphysema and vascular abnormalities in association with deregulated TGF-β signaling has also been shown in another mouse model with a deficiency in an ECM protein, Fibrillin-1, which is a model for Marfan syndrome [30]. Moreover, the combination of pulmonary emphysema and aortic aneurysms coinciding with upregulation of TGF-β signaling has also been observed in autosomal recessive cutis laxa syndrome caused by Fibulin-4 mutations [31]–[33]. The role for TGF-β in this process is further supported by the development of progressive airspace enlargement in Smad3 knockout mice, which are deficient for an intracellular regulator of the TGF-β pathway [34]. Overall, these data point to deregulated TGF-β signaling and ECM defects as common underlying factors for aortic and pulmonary abnormalities.

Expression analysis further revealed downregulation of the SERPINA1 gene, encoding for the serine protease inhibitor α-1 antitrypsin whose targets include elastase. Interestingly, overlapping gene expression profiles of our Fibulin-4 deficient mice with those of COPD patients revealed downregulation of SERPINA1 as a common denominator. As it is known that patients with α-1 antitrypsin deficiency develop COPD [22], [35] we explored the link between SERPINA1 and Fibulin-4. Pathway exploration in IPA revealed a direct link to MMP9, TGF-β deregulation and 15 other deregulated genes from our dataset. Although MMP9 itself was not overexpressed, molecular imaging showed that the MMP activity was gradually higher in Fibulin-4^+/R^ and Fibulin-4^R/R^ lungs. In line with the observed downregulation of SERPINA1, fluorescent imaging showed a gradual up-regulation of elastase in Fibulin-4^+/R^ and Fibulin-4^R/R^ lungs, which correlated with elastin fragmentation. The decreased expression of SERPINA1 may either be a direct effect of Fibulin-4 deficiency, or an indirect effect through its cleavage by MMP9[24]. This combination of increased protease activity and decreased antiprotease activity may account for the breakdown of alveolar walls, resulting in emphysema.

Another hallmark of adult Fibulin-4 mice was the inflammatory response in the lungs. Lungs of adult Fibulin-4^R/R^ mice already displayed pulmonary inflammation in a specific pathogen free environment, including influx of a wide range of inflammatory cells with elevated levels of the pro-inflammatory cytokine IL-1β, which coincided with the overexpression of genes involved in inflammatory pathways. In contrast, adult Fibulin-4^+/R^ animals did not exhibit pulmonary inflammation under baseline conditions, but displayed an enhanced respiratory inflammatory response upon LPS inhalation. These findings indicate that although inflammation may contribute to the progressive breakdown of alveolar walls in adult Fibulin-4^R/R^ mice, it is unlikely to be the primary causative factor in Fibulin-4^+/R^ mice. Conversely, ECM degradation by proteases is known to induce the release of bioactive fragments that may act as chemo-attractants for leukocytes and modulate the activity of resident immune cells [36]. Our data suggest that a mild Fibulin-4 deficiency induces disruption of the ECM, which subsequently predisposes to an enhanced inflammatory response with further breakdown of alveolar walls. This vicious circle is further exacerbated by the diminished antiprotease capacity of the lungs and ultimately results in the development of pulmonary emphysema. The Fibulin-4^R/R^ mouse can therefore provide as a model for adverse lung development, while the heterozygous Fibulin-4^+/R^ mouse may serve as a postnatal challenge model.

The traditional inflammatory model of COPD proposes that in susceptible patients cigarette smoking leads to inflammation, which subsequently induces loss of ECM and alveoli, resulting in airspace enlargement. We propose that genetic ECM defects are one of the initiating events contributing to this susceptibility, which are associated with a heightened inflammatory response to environmental triggers, such as microorganisms and smoking. Such a generalized genetic susceptibility to ECM degradation and secondary inflammation in combination with increased protease activity and decreased anti-protease activity might be the common pathophysiologic mechanism underlying the tissue destruction in both COPD and aneurysm formation. Genetic screening for mutations related to ECM defects may be a new strategy to identify people at risk for developing both aneurysms and COPD with age.

## Materials and Methods

### Clinical study

#### Patients

Consecutive patients undergoing elective open or endovascular surgery for aortic aneurysm, peripheral arterial disease, or carotid artery disease between 2002 and 2011 in the Erasmus MC, Rotterdam, were included. Patients with an aortic aneurysm (AA) were classified as aneurysmal disease. Patients with atherosclerotic peripheral arterial or carotid artery disease were classified as arterial occlusive disease (AOD). Patients treated with combined AA and symptomatic AOD, and patients with a genetic aneurysm syndrome like Marfan, Loeys-Dietz or vascular Ehlers-Danlos syndrome were excluded. The study complies with the declaration of Helsinki and was approved by the Institutional Review Board of the Erasmus Medical Center (permit number MEC-2011-510) in accordance with national and international guidelines. Our institutional review board waived the need for written informed consent from the participants since the data was obtained for clinical purpose, there was no intervention and there was a retrospective study design. Patient data were de-identified prior to analysis.

#### Clinical characteristics

Medical history was obtained from every patient, including the cardiovascular risk factors age, gender, body mass index (BMI), smoking status, hypertension (blood pressure ≥140/90 mmHg in non-diabetics, ≥130/80 mmHg in diabetics, or use of antihypertensive medication), hypercholesterolemia (low-density lipoprotein [LDL] cholesterol ≥3.5 mmol/L or use of lipid lowering medication), diabetes mellitus (fasting plasma glucose ≥7.0 mmol/L, non-fasting glucose ≥11.1 mmol/L, or use of anti-diabetic medication), and kidney disease (serum creatinine ≥2.0 mg/dl). Cardiovascular comorbidities were recorded, including congestive heart failure (defined as history of congestive heart failure), ischemic heart disease (defined as a history of angina pectoris, myocardial infarction, coronary revascularization, or presence of pathologic Q-waves on the electrocardiogram), cerebrovascular disease (defined as a history of ischemic/hemorrhagic stroke or transient ischemic attack). Prescription medications were recorded and included the use of statins, beta-blockers, renin-angiotensin system inhibitors, diuretics, and antiplatelet drugs. Serum concentrations of the inflammatory biomarker high-sensitivity C-reactive protein (hs-CRP) were measured prior to surgery using immunochemistry (Beckman Coulter, Woerden, the Netherlands).

#### Chronic obstructive pulmonary disease

The diagnosis and classification of COPD was made using spirometry, which was part of the routine preoperative workup and was obtained in 92% of COPD patients. COPD was defined as the presence of a forced expiratory volume in one second (FEV1) to forced vital capacity (FVC) ratio (FEV1/FVC) <0.70. In the presence of a FEV1/FVC ratio of <0.70, mild COPD was defined as a FEV1>80% of the predicted FEV1 (GOLDI), moderate COPD was defined as a FEV1 of 50–80% of the predicted FEV1 (GOLDII), and severe COPD was defined as a FEV1<50% of the predicted FEV1 (GOLDIII/IV)[37]. Patients without spirometry were classified based on the presence of pulmonary symptoms (i.e. cough, dyspnea, sputum) and the use of pulmonary medication.

#### Statistical analysis

Dichotomous data are presented as numbers and percentages. Continuous variables are presented as mean ± standard deviation or median and IQR when not normally distributed. Categorical data were analyzed with chi-square tests and continuous variables with ANOVA or Kruskal-Wallis tests. Multivariable binary logistic regression analysis was used to calculate odds for having COPD between AA and AOD. Adjustments were made for age, gender, BMI, congestive heart failure, ischemic heart disease, cerebrovascular disease, kidney disease, diabetes mellitus, hypertension, hypercholesterolemia, smoking, statins, beta-blockers, renin-angiotensin system inhibitors, diuretics, antiplatelets, and hs-CRP. Furthermore, we performed a propensity score to adjust for the possibility of receiving a pulmonary function test prior to surgery. Covariates were chosen on the bases of biological plausibility. For all tests, a p-value <0.05 (two-sided) was considered significant. All analyses were performed using IBM SPSS Statistics version 20.0 (SPSS Inc., Chicago, IL, USA).

### Experimental study

#### Animals

Fibulin-4 animals were generated as previously described [14]. All mice used were bred in a C57BI/6J background and were kept in individually ventilated cages to keep animals consistently micro-flora and disease free. To avoid stress-related vascular injury, mice were earmarked and genotyped 4 weeks after birth. Mice used were either newborn or adult (110±10 days). Adult mice were challenged by a single intratracheal injection with either 80 µl ultra-pure, sterile Lipopolysaccharide (LPS) 1 mg/ml from *E. coli* Serotype R515 (Alexis Corporation Switzerland) or 80 µl PBS (Lonza). Animals were housed at the Animal Resource Centre (Erasmus University Medical Centre), which operates in compliance with the “Animal Welfare Act” of the Dutch government, using the “Guide for the Care and Use of Laboratory Animals” as its standard. As required by Dutch law, formal permission to generate and use genetically modified animals was obtained from the responsible local and national authorities. An independent Animal Ethics Committee of the Erasmus Medical Center (Stichting DEC Consult) approved these studies (permit number 139-10-12 and 139-12-02), in accordance with national and international guidelines.

#### Quantitative real time PCR

RNA was isolated using the RNeasy minikit from Qiagen according to the provided protocol and synthesized to cDNA with the RevertAid H Minus First Strand cDNA Synthesis Kit according to the provided instructions. Quantitative Real-Time PCR was performed using Maxima SYBR Green qPCR Master Mix 2x (Fermentas) also according to the provided protocol. Reactions were performed in triplicates per gene for each sample. The primers used for Fibulin-4, Gapdh and Hprt (Invitrogen) are indicated in Table S7. Product specificity was determined by melting curve analysis and gel electrophoresis. The average Ct values of the triple reactions were calculated for each gene according to cell type. The relative gene expression level was calculated by the following formula for each gene:

Relative gene expression level = 2^(Ct control–Ct sample) gene^/2^(Ct control–Ct sample) housekeeping gene.^

The levels of fold-change for each gene were calculated by dividing the relative gene expression levels in Fibulin-4^+/R^ or Fibulin-4^R/R^ lungs to the relative gene expression levels in wild type lungs.

#### Whole body plethysmography

Conscious mice were placed in a single-chamber, whole body, plethysmograph (Emka Technologies, Paris, France) as described previously [38]. After an adaptation period of 9 minutes (acclimatization), Peak Inspiration Flow (PIF) and Peak Expiration Flow (PEF) were measured in 6 time blocks of 3 minutes. Differences in PIF and PEF indicate differences in inspiration and expiration strength.

#### Lung morphometry

A random selection of images of HE stained alveoli were obtained with the Leica DFC280DFC480 (Aristoplan) with a magnification of 10x. Large airways and vessels were generally avoided. Next, alveolar airspace size quantification was performed according to the fully automated D_2_ method as described in Jacob RE et. al, where it was compared to the semi-automated mean linear intercept measurements, and turned out to be more sensitive and specific for subtle airspace enlargement expected to be found in mild or early stage emphysema [39]. All images were converted to grayscale before performing the analysis. Fuzzy-c-means clustering with simultaneous correction of potential luminance inhomogeneity was applied to each image for pre-segmenting it into two classes: the foreground and the background. The final segmentation was obtained by the graph-cut method with the energies given by the class membership functions calculated on the previous step. The resulting foreground was split into separate compartments corresponding to the connected components belonging to this class; see Figure S1 and the accompanying legend for an illustration. Vector of the compartment sizes obtained in such a way was converted from pixels to micrometers. For each of the vectors we calculated the D_2_ measure [33], an index based on the equivalent diameters of airspaces and by incorporating higher moment factors from the airspace diameter distributions, where enlarged airspaces are weighed more heavily. This measure is useful to detect early or mild emphysema. The compartments whose sizes were less than 138 µm were disregarded according to the threshold previously reported in [39].

#### Histological analysis and immunohistochemistry

For the lung morphometry procedure, mice were euthanized with a lethal dose of pentobarbital (60 mg/ml, 0.1–1.5 ml per mouse according to weight). Lung lobes were excised and the left lobe was pressure fixed through the bronchi at a pressure of 25 cm H_2_O with 4% paraformaldehyde (PFA), and fixed overnight at 4°C before paraffin embedding. Lungs from newborn mice were immersion fixed. The 5-µm sections were prepared from the paraffin embedded lungs and put on Superfrost Ultra plus slides (Menzel-Glaser). For the morphometric analysis paraffin sections of the lungs were stained with Haematoxylin-Eosin (HE).

For histological analysis 100-day-old female mice were dissected. Mice were euthanized by CO_2_-inhalation. After opening thorax and abdomen, mice were fixed by perfusion fixation through the left ventricle, with PBS and 4% paraformaldehyde (PFA). Organ weights were determined and macroscopic abnormalities noted. Organs and tissues were fixed in 4% PFA. Lungs and aortas were dehydrated through the histokinette processor (Microm), and paraffin embedded, after which 5-µm sections were prepared.

Lungs and aortas were stained with HE for general pathology and Resorcin-Fuchsin (Elastin von Gieson) for elastin structure. For immunohistochemical analyses, sections were emerged in 3% H_2_O_2_ in PBS to inhibit endogenous peroxidase. Antigen retrieval was performed by boiling slides in 10 mM citrate buffer, pH 6.0, at 600 W for 15 minutes in a microwave for TTF-1 and CC10 staining, 100 mM Tris 10 mM EDTA buffer, pH 9.0, at 300 W for 20 minutes for pSmad-2 staining, or with pronase treatment for α-SMA. Slides were first blocked in 5% Bovine Serum Albumin (BSA) in PBS and 0.5% Tween (and 5% Protifar in PBS and 0.025% Triton X-100 for pSmad-2), and incubated with the primary antibodies overnight at 4°C; TTF-1 (1∶250 mouse monoclonal Ab-1 Clone 8G7G3/1 Thermo Fisher Scientific), CC10 (1∶100 goat Ab (T-18): sc-9772 Santa Cruz Biotechnology), Anti-Human Smooth Muscle Actin (1∶250 mouse, clone 1A4 Biogenex Laboratories Inc.), and pSmad-2 (1∶100 monoclonal Rabbit anti-pSmad2 (S465|467 (138D4) Cell Signaling). The next day slides were incubated with Horse Radish Peroxidase (HRP) labelled secondary antibodies (1∶100 DAKO) and avidin-biotinylated secondary antibodies (Vectastain Universal Elite ABC kit Vector Laboratories) for pSmad-2. DAB chromogen (DAKO Liquid Dab substrate-chromogen system) was used as substrate and slides were counterstained with haematoxylin.

Immunohistochemical stainings for inflammatory cells were performed in a half-automatic stainer (Sequenza, Amsterdam, the Netherlands). Acetone-fixed slides were blocked in diluted normal goat serum (CLB, Amsterdam, the Netherlands) and stained against mouse CD3 (1∶10 rat monoclonal antibodies KT3 AbD Serotec) and against mouse CD11c (1∶20 hamster antibodies N418 Ebioscience). Primary antibodies were revealed by incubation with diluted appropriate secondary antibodies coupled to alkaline phosphatase for 30 min. Slides were subsequently incubated with New Fuchsin substrate for alkaline phosphatase conjugates. Finally, the sections were counterstained with Gills triple-strength haematoxylin and mounted in VectaMount (Brunschwig, Amsterdam).

#### Micro-array hybridizations

RNA was isolated using the RNeasy minikit from Qiagen with the provided protocol and delivered to the department of Biomics, Erasmus MC. Synthesis of double stranded cDNA and biotin labelled cRNA was performed according to the instructions of the manufacturer (Affymetrix). Fragmented cRNA was hybridized to Mouse Genome 430 V2.0 arrays, using a hybridization Oven 640 (Affymetrix), washed and subsequently scanned on a GeneChip Scanner 3000 (Affymetrix). To examine the quality of the various arrays, several bioinformatic R packages (including affyQCreport and affyPLM) were run starting from the raw CEL data files. All created plots, including RNA degradation, RLE and NUSE plots indicated a high quality of all samples and an overall comparability, except for one sample (Fibulin-4^R/R^ newborn lung), which was excluded from further analysis. Raw intensity values of all samples were normalized by robust multichip analysis normalization (background correction and quantile normalization) using Partek version 6.4 (Partek Inc., St. Louis, MO). The normalized data file was transposed and imported into OmniViz version 6.0.1 (Biowisdom, Ltd., Cambridge, UK) for further analysis. For each probe set, the geometric mean of the hybridization intensities of all samples was calculated. The level of expression of each probe set was determined relative to this geometric mean and ^2^log transformed. The geometric mean of the hybridization signal of all samples was used to ascribe equal weight to gene expression levels with similar relative distances to the geometric mean. Differentially expressed genes were identified using ANOVA (Partek) and SAM (Omniviz). The cut-off value for significantly expressed genes was FDR 10% for adult Fibulin-4^R/R^ lungs compared to Fibulin-4^+/+^ lungs. Functional and network analysis was done using Ingenuity Pathway Analysis (IPA; Ingenuity Systems, www.ingenuity.com, Mountain View, CA). Ingenuity pathway analysis is a web-based software application that enables to analyze and integrate data derived from gene expression microarrays into biological networks and pathways. All Ingenuity products leverage the Ingenuity Knowledge Base, which houses biological and chemical relationships extracted from the scientific literature.

Significantly expressed genes from the adult Fibulin-4^R/R^ to Fibulin-4^+/+^ lungs comparison were compared to COPD-associated genes in Ingenuity and a list of literature based genes associated with COPD. CEL files from GEO dataset GSE8581 were obtained and were analyzed following the above described procedures. The GEO dataset GSE8581 consisted of 15 COPD cases, with a predicted FEV1<70% and FEV1/FVC<0.7, and 18 control cases, with a predicted FEV1>80% and FEV1/FVC>0.7. Subjects were undergoing surgical resection of a suspected lung tumor and tissue for this dataset was derived from histologically normal lung tissue distant from the tumor margin [40]. Cut-off values for significantly expressed genes were FDR 30% and 1.5-fold. Comparison to significantly expressed genes from the adult Fibulin-4^R/R^ to Fibulin-4^+/+^ lungs comparison was done using IPA.

**Preparation of cell suspensions, flow cytometry and ELISA**. Broncho Alveolar Lavage (BAL) was performed with 3 times 1 ml of Ca^2+^- and Mg^2+^-free PBS, containing 10 mM EDTA. Furthermore, lungs were enzymatically digested using collagenase type III (Worthington) for 1 hour at 37°C, followed by washing and filtering. Cell suspensions were stained with antibodies specific for F4/80-Fitc, MHC class II-PE, CD11c-PeTexasRed, CD3-PECy5, CD19-APCCy7, CD25-APC and GR-1-PECy7 (Becton Dickinson or eBiosciences). Nonspecific binding to Fc-receptors was blocked by incubation with 2.4G2 antibodies, and DAPI (Invitrogen) was used as life/dead marker. Acquisitions were performed on an LSRII flow cytometer (Becton Dickinson) and data were analyzed by FlowJo (Treestar, Costa Mesa, CA) software. Supernatants of BAL fluid were stored for ELISA. BAL fluid cytokines were measured by commercially available specific ELISA systems for IL-6, KC, MCP-1, TARC, IL-10, IL-12, IL-1β, TNF-α, IFN-gamma and IL-17 according to the manufacturers’ instructions. In a separate set of experiments, flow cytometric analyses of BAL samples and pulmonary cell suspensions were performed 18 or 72 hours after a single intratracheal injection with either sterile lipopolysaccharide (LPS) 1 mg/ml in PBS or PBS alone in adult (100-days-old) Fibulin-4^+/R^ and Fibulin-4^+/+^ mice.

#### Western blot analysis

Western blot analysis was performed as described before [41]. In short, equal amounts of lung tissue homogenates (40 ug) were separated under reducing conditions on 10% SDS-PAGE. Proteins were transferred to nitrocellulose membranes (Whatman, Germany) and blocked with 5% milk. After washing, membranes were incubated with rabbit anti-phosphorylated Smad2 (Cell Signaling Technologies, USA) and rabbit anti-phosphorylated Smad3, kindly provided by Dr. E. Leof, Mayo Clinic, Rochester, MN, USA followed by HRP labelled secondary antibodies (GE Healthcare) and detection with a chemiluminiscent substrate (Pierce). Afterwards membranes were stripped and reprobed with anti Smad2/3 antibodies (BD biosciences), β-actin (Sigma) or GAPDH (Millipore) as a loading control.

#### Fluorescence imaging

We used vascular fluorescent mediated tomography (FMT) imaging with near-infrared fluorescent protease activatable probes as previously described [15], [42]. Open chest FMT imaging of fibulin-4 mice was performed using an FMT 2500 system (Perkin Elmer Inc.) at 680- and 750-nm excitation and emission wavelengths, respectively, at five hours after tail vein injection of 4 nmol of Neutrophil Elastase 680 FAST and 2 nmol of MMPsense 750 FAST (Perkin Elmer Inc.). Mice with open chests were fixed into the portable animal imaging cassette that lightly compressed the mouse between optically translucent windows. The FMT 2500 quantitative tomography software was then used to calculate 3D fluorochrome concentration distribution of Neutrophil Elastase 680 FAST and MMPsense 750 FAST.

After open chest fluorescence imaging, complete lungs were harvested and fluorescence was quantified using the FMT 2500 or Odyssey imaging systems (LI-COR Inc.). Near-infrared images were obtained in the 680- and 700-nm channels, respectively.

#### Statistical analysis

Data are presented as mean ± SEM. Statistical analysis for lung morphometry was performed using the Kolmogorov-Smirnov test. The Kruskal-Wallis one-way ANOVA was used to determine any significant differences between groups. The nonparametric Mann-Whitney U-test was performed to analyze the specific sample pairs for significant differences. A p-value of <0.05 was considered to indicate a significant difference between groups. All analyses were performed using IBM SPSS Statistics version 20.0 (SPSS Inc., Chicago, IL, USA).

## Supporting Information

Figure S1 **Larger alveolar airspaces in newborn Fibulin-4^R/R^ lungs (A) and adult Fibulin-4^+/R^ and Fibulin-4^R/R^ lungs (B).** To quantify and compare the sizes of the alveolar airspaces, the compartments from the different alveolar airspaces were segmented on the HE images according to the method described in the “Lung morphometry” section of the Material and Methods section. Each segmented compartment was given a different color as shown and subsequently quantified as described. Magnification 10x. Scale bar 100 µm. (C, D) Comparison of the architecture of the aortic wall in Fibulin-4^+/+^, Fibulin-4^+/R^, and Fibulin-4^R/R^ mice used for alveolar airspace analysis in Figure 3C. Haematoxylin- eosin (HE) staining of cross-sections from 120 day-old mice (C). Aberrations in elastic laminae in Fibulin-4^R/R^ mice, consisting of a fragmented and disorganized appearance of elastin in the medial layers of the aorta (D).
(TIF)

Figure S2 **Similar cell structures in wild type and Fibulin-4 knockdown lungs.** Stainings for (A) respiratory epithelial cells with TTF-1, (B) Clara cells with CC10 and (C) smooth muscle cells with α-SMA show similar cell structures in Fibulin-4^+/+^ (n = 3), Fibulin-4^+/R^ (n = 3) and Fibulin-4^R/R^ (n = 2) lungs. Magnification 10x. Scale bar 100 µm.
(TIF)

Figure S3 **LPS infection induces infiltration of inflammatory cells in the alveolar compartments.** Quantification of immune cells in alveolar compartments shows increased CD3+ cells after 72 hours of LPS exposure in Fibulin-4^+/R^ (n = 4) as compared to Fibulin-4^+/+^ lungs (n = 4, *p<0.05).
(TIF)

Table S1 **Clinical characteristics of patients with descending thoracic aortic aneurysm (TAA) or abdominal aortic aneurysm (AAA).**
(DOCX)

Table S2 **COPD in patients with descending thoracic aortic aneurysm (TAA) or abdominal aortic aneurysm (AAA).**
(DOCX)

Table S3 **Association between COPD and aneurysmal disease.**
(DOCX)

Table S4 **The most significantly down-regulated genes in lungs of adult Fibulin-4^R/R^ mice.**
(DOCX)

Table S5 **Over-expressed canonical pathways, based on IPA, in lungs of adult Fibulin-4^R/R^ mice (p<0.05).**
(DOCX)

Table S6 **Deregulated TGF-β pathway genes in adult and newborn Fibulin-4 deficient lungs compared to Fibulin-4^+/+^ lungs (p<0.05).**
(DOCX)

Table S7 **Primers used for quantitative real time PCR. Forward and reverse primers are displayed for each gene from 5′ to 3′.**
(DOCX)
