# Supplementary figures and images for: Extracellular Matrix Defects in Aneurysmal Fibulin-4 Mice Predispose to Lung Emphysema
Source: PLoS One. 2014 Sep 25;9(9):e106054. doi: 10.1371/journal.pone.0106054 (PMC4177830; doi:10.1371/journal.pone.0106054)

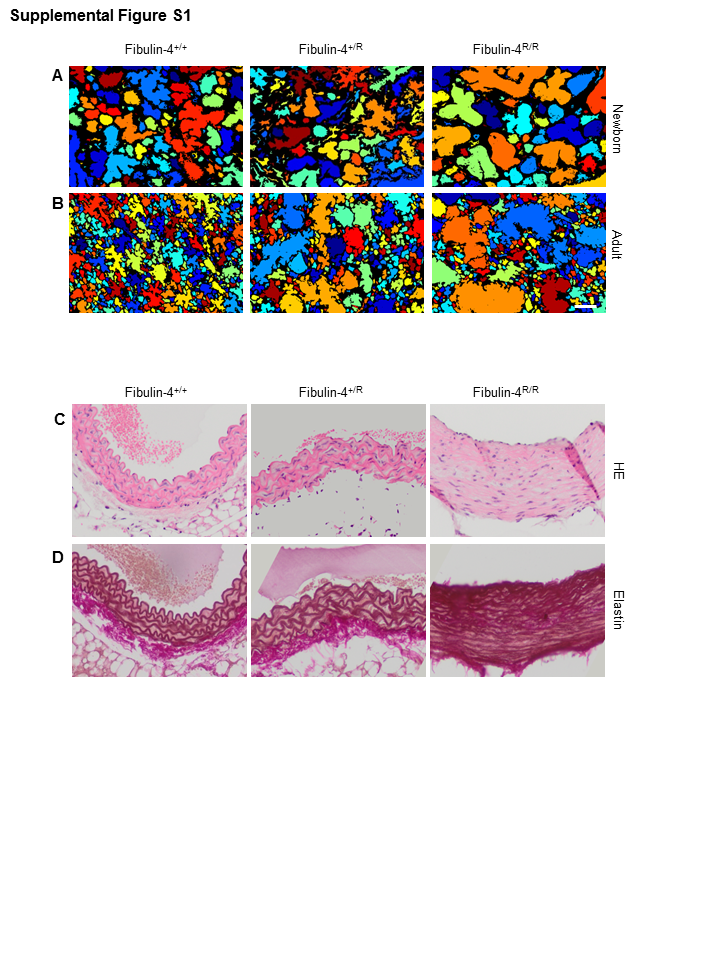

Supplement: Figure S1 — Larger alveolar airspaces in newborn Fibulin-4R/R lungs (A) and adult Fibulin-4+/R and Fibulin-4R/R lungs (B). To quantify and compare the sizes of the alveolar airspaces, the compartments from the different alveolar airspaces were segmented on the HE images according to the method described in the “Lung morphometry” section of the Material and Methods section. Each segmented compartment was given a different color as shown and subsequently quantified as described. Magnification 10x. Scale bar 100 µm. (C, D) Comparison of the architecture of the aortic wall in Fibulin-4+/+, Fibulin-4+/R, and Fibulin-4R/R mice used for alveolar airspace analysis in Figure 3C. Haematoxylin- eosin (HE) staining of cross-sections from 120 day-old mice (C). Aberrations in elastic laminae in Fibulin-4R/R mice, consisting of a fragmented and disorganized appearance of elastin in the medial layers of the aorta (D). (TIF) [file pone.0106054.s001.tif]

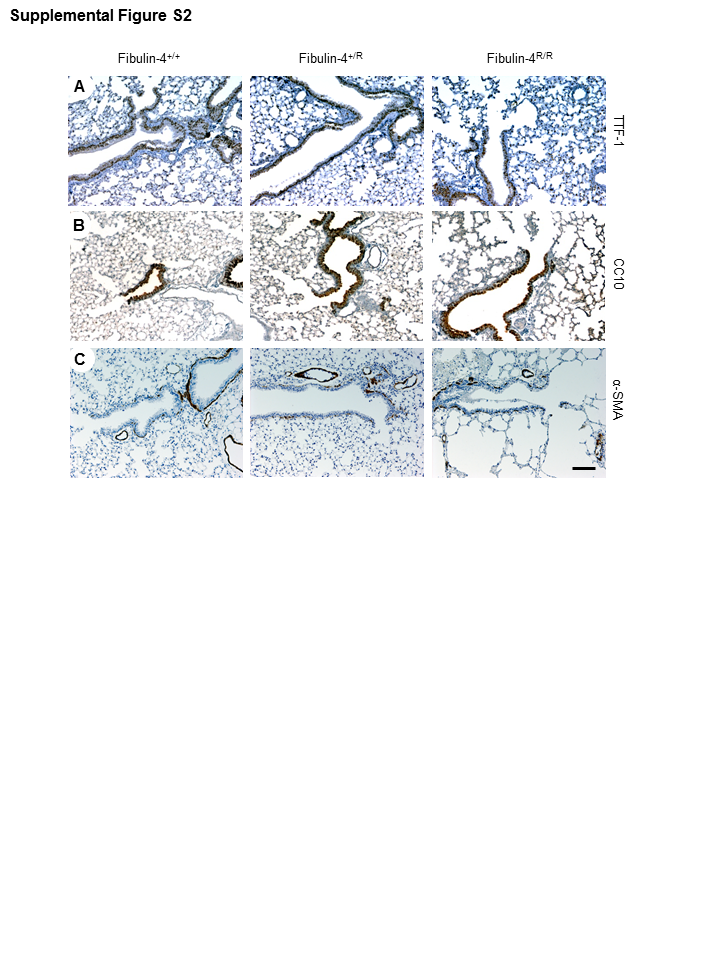

Supplement: Figure S2 — Similar cell structures in wild type and Fibulin-4 knockdown lungs. Stainings for (A) respiratory epithelial cells with TTF-1, (B) Clara cells with CC10 and (C) smooth muscle cells with α-SMA show similar cell structures in Fibulin-4+/+ (n = 3), Fibulin-4+/R (n = 3) and Fibulin-4R/R (n = 2) lungs. Magnification 10x. Scale bar 100 µm. (TIF) [file pone.0106054.s002.tif]
