# Supplementary material for: Extracellular Matrix Defects in Aneurysmal Fibulin-4 Mice Predispose to Lung Emphysema
Source: PLoS One. 2014 Sep 25;9(9):e106054. doi: 10.1371/journal.pone.0106054 (PMC4177830; doi:10.1371/journal.pone.0106054)
Supplement: Table S1 — Clinical characteristics of patients with descending thoracic aortic aneurysm (TAA) or abdominal aortic aneurysm (AAA). (DOCX) [file pone.0106054.s004.docx]

**SUPPLEMENTAL TABLES**

*Supplemental Table S1 – Clinical characteristics of patients with descending thoracic aortic aneurysm (TAA) or abdominal aortic aneurysm (AAA).*

|  | TAA | AAA | P-value |
| --- | --- | --- | --- |
|  | n=62 | n=552 |  |
| Baseline characteristics |  |  |  |
| Male gender (%) | 37 (59.7) | 488 (88.4) | <0.001 |
| Age (years ± SD) | 69.0 ± 8.7 | 71.6 ± 7.6 | 0.013 |
| Body mass index (kg/m2, mean ± SD) | 25.2 ± 4.1 | 26.2 ± 3.8 | 0.054 |
| Cardiovascular comorbidities (%) |  |  |  |
| Congestive heart failure | 4 (6.5) | 62 (11.2) | 0.264 |
| Ischemic heart disease | 16 (25.8) | 256 (46.4) | 0.002 |
| Cerebrovascular disease | 5 (8.1) | 84 (15.2) | 0.129 |
| Cardiovascular risk factors (%) |  |  |  |
| Kidney disease | 4 (6.5) | 90 (16.3) | 0.041 |
| Diabetes mellitus | 7 (11.3) | 96 (17.4) | 0.223 |
| Hypertension | 44 (71.0) | 364 (65.9) | 0.340 |
| Hypercholesterolemia | 53 (85.5) | 481 (87.1) | 0.714 |
| Smoking – current | 22 (35.5) | 214 (38.8) | 0.776 |
| Smoking – ever | 45 (72.6) | 428 (77.5) | 0.379 |
| Medication (%) |  |  |  |
| Statins | 44 (71.0) | 402 (72.8) | 0.986 |
| Beta-blockers | 48 (77.4) | 483 (87.5) | 0.087 |
| Renin-angiotensin system inhibitors | 31 (50.0) | 240 (43.5) | 0.235 |
| Diuretics | 14 (22.6) | 124 (22.5) | 0.890 |
| Antiplatelets | 27 (43.5) | 326 (59.1) | 0.033 |
