# Supplementary material for: Extracellular Matrix Defects in Aneurysmal Fibulin-4 Mice Predispose to Lung Emphysema
Source: PLoS One. 2014 Sep 25;9(9):e106054. doi: 10.1371/journal.pone.0106054 (PMC4177830; doi:10.1371/journal.pone.0106054)
Supplement: Table S2 — COPD in patients with descending thoracic aortic aneurysm (TAA) or abdominal aortic aneurysm (AAA). (DOCX) [file pone.0106054.s005.docx]

*Supplemental Table S2 – COPD in patients with descending thoracic aortic aneurysm (TAA) or abdominal aortic aneurysm (AAA).*

|  | TAA | AAA | P-value |
| --- | --- | --- | --- |
|  | n=62 | n=552 |  |
| Total COPD | 31 (50.0) | 228 (41.3) | 0.189 |
| GOLD I (%) | 12 (19.4) | 86 (15.6) |  |
| GOLD II (%) | 14 (22.6) | 103 (18.7) | 0.625 |
| GOLD III/IV (%) | 5 (8.1) | 39 (7.1) |  |
