# Supplementary material for: Extracellular Matrix Defects in Aneurysmal Fibulin-4 Mice Predispose to Lung Emphysema
Source: PLoS One. 2014 Sep 25;9(9):e106054. doi: 10.1371/journal.pone.0106054 (PMC4177830; doi:10.1371/journal.pone.0106054)
Supplement: Table S3 — Association between COPD and aneurysmal disease. (DOCX) [file pone.0106054.s006.docx]

*Supplemental Table S3 – Association between COPD and aneurysmal disease.*

|  | Univariable | | | | Multivariable^*^ | | |
| --- | --- | --- | --- | --- | --- | --- | --- |
|  | odds ratio | 95%CI | P-value | odds ratio | | 95%CI | P-value |
| No COPD | 1.00 |  |  | 1.0 | |  |  |
| COPD | 2.08 | [1.66 – 2.61] | <0.001 | 1.56 | | [1.16– 2.10] | 0.003 |
| Mild COPD | 2.34 | [1.67 – 3.28] | <0.001 | 1.66 | | [1.08 – 2.57] | 0.022 |
| Moderate COPD | 1.83 | [1.36 – 2.46] | <0.001 | 1.40 | | [0.97 – 2.04] | 0.075 |
| Severe COPD | 2.38 | [1.47 – 3.86] | <0.001 | 1.63 | | [0.85 – 3.15] | 0.142 |

^*^ Adjusted for: age, gender, BMI, congestive heart failure, ischemic heart disease, cerebrovascular disease, kidney disease, diabetes mellitus, hypertension, hypercholesterolemia, smoking, statins, beta-blockers, renin-angiotensin system inhibitors, diuretics, antiplatelets and hs-CRP.
