# Supplementary material for: Extracellular Matrix Defects in Aneurysmal Fibulin-4 Mice Predispose to Lung Emphysema
Source: PLoS One. 2014 Sep 25;9(9):e106054. doi: 10.1371/journal.pone.0106054 (PMC4177830; doi:10.1371/journal.pone.0106054)
Supplement: Table S4 — The most significantly down-regulated genes in lungs of adult Fibulin-4R/R mice. (DOCX) [file pone.0106054.s007.docx]

*Supplemental Table S4 - The most significantly down-regulated genes in lungs of adult Fibulin-4^R/R^ mice. The genes are indicated with their ratios compared to Fibulin-4^+/+^ mice and the process involved.*

| Top down-regulated genes | | |
| --- | --- | --- |
| Genes | Ratio | Function |
| Efemp2 | 3.56 | Extracellular matrix protein |
| Myrip | 2.25 | Melanosome transport |
| Krtap17-1 | 2.20 | Interfilamentous matrix proteins |
| Fam107a | 2.10 | Tumor development |
| Gdpd2 | 1.87 | Hydrolyzes glycerophosphoinositol |
| Dcdc2 | 1.82 | Microtubule polymerization |
| Mus81 | 1.82 | Endonuclease |
| Acot1 | 1.78 | Catalyze the hydrolysis of acyl-CoAs |
| Apob | 1.76 | LDL apolipoprotein |
| Slc15a2 | 1.74 | Proton-coupled peptide transporter in small intestine |
| Hspa4l | 1.70 | Chaperone activity |
| Galntl2 | 1.69 | Oligosaccharide biosynthesis |
| Hist2h3c | 1.67 | Nucleosome structure |
| Hmgcs2 | 1.67 | Mitochondrial enzyme involved in ketogenesis |
| Slc6a2 | 1.65 | Neurotransmitter transporter |
| Lonrf3 | 1.65 | Protein-protein and protein-DNA interactions |
| Ccl20 | 1.65 | Chemotactic factor that attracts lymphocytes and slightly neutrophils |
| Msc | 1.63 | Downstream target of the B-cell receptor signal transduction pathway |
| Fmo3 | 1.58 | Oxidative metabolism |
