# Supplementary material for: Extracellular Matrix Defects in Aneurysmal Fibulin-4 Mice Predispose to Lung Emphysema
Source: PLoS One. 2014 Sep 25;9(9):e106054. doi: 10.1371/journal.pone.0106054 (PMC4177830; doi:10.1371/journal.pone.0106054)
Supplement: Table S5 — Over-expressed canonical pathways, based on IPA, in lungs of adult Fibulin-4R/R mice (p<0.05). (DOCX) [file pone.0106054.s008.docx]

*Supplemental Table S5 - Over-expressed canonical pathways, based on IPA, in lungs of adult Fibulin-4^R/R^ mice (p<0.05). Genes associated with these pathways are shown with their log ratio changes compared to Fibulin-4^+/+^ lungs.*

| Canonical pathways | P-value | Involved genes (log ratio) |
| --- | --- | --- |
| Cholesterol Biosynthesis | 6.27 10^-5^ | HSD17B7, MSMO1, SC5DL, CYP51A1 |
| Zymosterol Biosynthesis | 1.09 10^-4^ | HSD17B7, MSMO1, CYP51A1 |
| Tumoricidal Function of Hepatic Natural Killer Cells | 8.66 10^-3^ | ENDOG, PRF1, ITGAL |
| Aldosterone Signaling in Epithelial Cells | 1.03 10^-2^ | HSPA12B, HSPB2, HSP90AA1, PLCL2, DNAJB2, HSPA2, HSPA4L, PRKCB |
| Sonic Hedgehog Signaling | 1.08 10^-2^ | STK36, PTCH1, HHIP |
| Granzyme A Signaling | 2.18 10^-2^ | GZMA, PRF1 |
| Acyl-CoA Hydrolysis | 2.18 10^-2^ | ACOT2, ACOT1 |
| Cytotoxic T Lymphocyte-mediated Apoptosis of Target Cells | 3.01 10^-2^ | PRF1, TRA, BCL2 |
| Glucocorticoid Receptor Signaling | 3.15 10^-2^ | FKBP4, SLPI, TGFB2, HSP90AA1,CCL5, CD163, HSPA2, TRA, BCL2 |
| Granzyme B Signaling | 3.24 10^-2^ | ENDOG, PRF1 |
| Hepatic Fibrosis / Hepatic Stellate Cell Activation | 3.32 10^-2^ | MYH10, MYH14, IL10RA, TGFB2, CCL5, BCL2 |
| Atherosclerosis Signaling | 4.19 10^-2^ | APOB, MMP3, SERPINA1, APOC2, ITGA4 |
| p38 MAPK Signaling | 4.29 10^-2^ | HSPB2, MAPT, HIST2H3C, TGFB2, MEF2C |
| Reelin Signaling in Neurons | 4.17 10^-2^ | MAPT, HCK, ITGAL, ITGA4 |
| LXR/RXR Activation | 4.89 10^-2^ | APOB, VTN, SERPINA1, APOC2, CYP51A1 |
