# Supplementary material for: Extracellular Matrix Defects in Aneurysmal Fibulin-4 Mice Predispose to Lung Emphysema
Source: PLoS One. 2014 Sep 25;9(9):e106054. doi: 10.1371/journal.pone.0106054 (PMC4177830; doi:10.1371/journal.pone.0106054)
Supplement: Table S6 — Deregulated TGF-β pathway genes in adult and newborn Fibulin-4 deficient lungs compared to Fibulin-4+/+ lungs (p<0.05). (DOCX) [file pone.0106054.s009.docx]

*Supplemental Table S6 - Deregulated TGF-β pathway genes in adult and newborn Fibulin-4 deficient lungs compared to Fibulin-4^+/+^ lungs (p<0.05).*

| Comparison | Deregulated TGF-β pathway genes | Gene symbol | Fold change |
| --- | --- | --- | --- |
| Adult Fibulin-4^R/R^ versus Fibulin-4^+/+^ | Transforming growth factor β2 | Tgfb2 | ↑ 1.46 |
|  | Activin A receptor type 2b | Acvr2b | ↓ 1.24 |
| Adult Fibulin-4^+/R^ versus Fibulin-4^+/+^ | SMAD specific E3 ubiquitin protein ligase 1 | Smurf1 | ↓ 1.23 |
| Newborn Fibulin-4^R/R^ versus Fibulin-4^+/+^ | Protein inhibitor of activated STAT 4 | Pias4 | ↓ 1.25 |
| Newborn Fibulin-4^+/R^ versus Fibulin-4^+/+^ | None | - | - |
