# Supplementary material for: Extracellular Matrix Defects in Aneurysmal Fibulin-4 Mice Predispose to Lung Emphysema
Source: PLoS One. 2014 Sep 25;9(9):e106054. doi: 10.1371/journal.pone.0106054 (PMC4177830; doi:10.1371/journal.pone.0106054)
Supplement: Table S7 — Primers used for quantitative real time PCR. Forward and reverse primers are displayed for each gene from 5′ to 3′. (DOCX) [file pone.0106054.s010.docx]

*Supplemental Table S7 - Primers used for quantitative real time PCR. Forward and reverse primers are displayed for each gene from 5’ to 3’.*

| Genes | Forward primers | Reverse primers |
| --- | --- | --- |
| *Fibulin-4* | 5’-GGGTTATTTGTGTCTGCCTCG-3’ | 5’-TGGTAGGAGCCAGGAAGGTT-3’ |
| *Gapdh* | 5’-ACCACAGTCCATGCCATCAC-3’ | 5’-TCCACCACCCTGTTGCTGTA-3’ |
| *Hprt* | 5’-CGAAGTGTTGGATACAGGCC-3’ | 5’-GGCAACATCAACAGGACTCC-3’ |
